# New insights into the retinal microstructure-diurnal activity relationship in the African five-lined skink (*Trachylepis quinquetaeniata*) (Lichtenstein, 1823)

**DOI:** 10.1186/s40851-023-00205-w

**Published:** 2023-03-18

**Authors:** Neveen E. R. El-Bakary, Mohamed A. M. Alsafy, Samir A. A. El-Gendy, Samar M. Ez Elarab

**Affiliations:** 1 Department of Zoology, Faculty of Science, Damietta University, Damietta, Egypt; 2 Anatomy and Embryology Department, Faculty of Veterinary Medicine, Alexandria University, Abees 10Th, Alexandria, Egypt; 3 Histology and Cytology Department, Faculty of Veterinary Medicine, Alexandria University, Alexandria, Egypt

**Keywords:** Retina, Ultrastructure, Photoreceptors, Reptilian, Diurnal

## Abstract

**Background:**

The retinae of diurnal vertebrates have characteristics. Most lizards are strictly diurnal, and their retinal morphology is still unknown.

**Materials and methods:**

The retina of the African five-lined skink (*Trachylepis quinquetaeniata*) was studied using light and transmission electron microscopy.

**Results:**

The retina's ten layers were all detected. The inner nuclear layer was the thickest by an average of 67.66 μm, and the inner plexiform layer was 57.564 μm. There were elliptical, long cylindrical, and spherical melanosomes (small and large) in the pigment epithelial layer of the retina. The cylindrical melanosomes had a large area on the lateral surfaces of cones to increase light scatter absorption. The photoreceptor layer of the retina had cones only. There were single and double cones, with the double cones consisting of principal and accessory cones. The cones had inner and outer segments separated by oil droplets. A spherical paraboloid body existed between the limiting membrane and the ellipsoid. All single cones had a paraboloid, and double cones had a large paraboloid in the accessory cone. The presence of paraboloids and large ellipsoids with mitochondria of varying sizes may have helped focus the light on cone segments.

**Conclusion:**

The African five-lined skink's eye was light-adapted due to a variety of retinal specializations related to the demands of its diurnal lifestyle in its environment.

## Introduction

The African five-lined skink (*Trachylepis quinquetaeniata*), also known as rainbow mabuya, is a member of the Lygosominae subfamily and can be found in both rocky and grassland habitats. The species is found in Egypt and southern Africa. It has been present all over Africa and is an invasive species in Florida. They usually build their nests in trees [1, 2].

*Trachylepis quinquetaeniata* is a diurnal skink that lives in large groups and may dorm colonies [3]. The new lizard *Trachylepis quinquetaeniata* was observed on April 4 under sunny skies at 25 °C and 57% humidity and on June 20 at 32 °C and 66% humidity [2]. The Gekkonidae family contains the most nocturnal lizard species [4].

The optical cells of vertebrates are either rods or cones, which are related to scotopic or photopic vision, and the discrepancy depends on some morphological structures of the many photoreceptor structures [5–8].

In bright light, many vertebrate species have sophisticated high visual severity, including retinal-special structures like high cone density, oil droplets, pigmentation, the fovea, and the retinal pit [9–11]. The fovea is absent in shinks [12, 13], and it is primarily composed of cones to correlate and enhance visual acuity [14].

Most vertebrates have rods and cones together in their retinae (duplex retinae), but the ratios of rods to cones vary based on habits and environments. Rods are the majority in the retinae of nocturnal species, whereas cones are the majority in those of diurnal species [12, 15–17].

Furthermore, the retinae of diurnal vertebrates are characterized by thick inner nuclear and inner plexiform layers due to the connectivity patterns of the retinal neurons. These retinae typically have regional specializations such as visual streaks, areas, or foveae, which have higher densities of the visual and ganglion cells than unspecialized regions [18].

Many reptiles, such as lizards of the genus Anolis, possess a compound visual system containing two morphologically different and spatially separated foveae [19]. An additional visual component was the oil droplets located in the cones. These are widespread among diurnal birds and reptiles [20] and act like individual filters of the photoreceptors, permitting extra acute visualization [19] and removing the flashy effect on the surface of the water [21]. The pigmentation of these structures seems important because when pigmented, they decrease the light spectrum range reaching the outer segments of the cones and stimulate the excitation of the specific opsin of the cone. Some cones possess only translucent oil droplets that increase how the light is held and maximize visual sensitivity [19].

Most lizards are strictly diurnal [7, 22]. Their retinae are usually devoid of rods and are distinguished by centrally located foveae. Iguanids, chameleonids, agamids, scincids, lacertids, anguids, pygopodids, and varanids appear to have pure-cone retinae [4, 23, 24]. The morphology of the retinae of most reptilian groups, particularly lizards, is unknown, as it is in other vertebrates, birds, and mammals [25]. Electron microscopic investigations revealing the ultrastructure of visual cells are rare except for those in the Gekkonidae [25].

The current study aimed to describe the histological and ultrastructural architecture of the retina of the African five-lined skink (*Trachylepis quinquetaeniata*), understand the retina's morphology and relationship to the lizard's lifestyle, and collect additional data on diurnal lizard vision.

## Material and methods

### Samples collection

Ten adult African five-lined skinks (*Trachylepis quinquetaeniata*) were collected from Abou Rawash-Giza for this study (Egypt). The lizards were euthanized via intramuscular injection of a lethal dose of Ketamine hydrochloride (200 mg/kg body mass; Ketalar®). The heads were removed after decapitation. Both eyes were freshly dissected and prepared for light and transmission electron microscopy.

### Light and transmission electron microscopy

The dissected retina dorsal and ventral to the optic nerve was cut into small pieces and fixed in a fixative solution of 2% formaldehyde, 1.25% glutaraldehyde, and 0.1 M sodium cacodylate buffer at pH 7.2 and 4 °C [26]. After fixation, the tissues were washed every 15 min in 0.1 M phosphate buffer for 2 h at 4 °C. Then the tissues were post-fixed in a 1% solution of phosphate-buffered osmium tetroxide (2% osmic acid, 5 mL, and phosphate buffer, 5 mL) for 2 h at room temperature. Then, they were dehydrated for 30 min in each of a series of ascending ethyl alcohol concentrations (30, 50, 70, 90, and 100% for 2 changes). After that, it was transferred to propylene oxide and left overnight in a propylene-epoxy Araldite combination. After that, epoxy Araldite was used to embed them [27]. Polymerization of the embedding mixture and the tissue blocks was done in an oven for five days as follows: at 35 ˚C for 24 h, at 45 ˚C for another 24 h, and lastly at 60 ˚C for 3 days. Semithin Sects. (1 µm) were first cut, stained with toluidine blue, and examined under a light microscope to select suitable areas for electron microscopy. The ultrathin Sects. (60–100 nm) were then cut with a glass knife and stained with uranyl acetate before being stained with lead citrate [27]. These sections were examined with a JEOL JSM-IT200 scanning electron microscope (JEOL Ltd. 3–1-2 Musashino, Akishima, Tokyo 196–8558, Japan) operating at the Faculty of Science, Alexandria University [28].

### Morphometric analysis

The obtained TEM images were analyzed by the ImageJ application to measure the thickness of the retina layers and the area and circumference of the melanosomes and droplet oil, and the mean and standard error were done by Microsoft Excel [29].

## Results

**Fig. 1:**
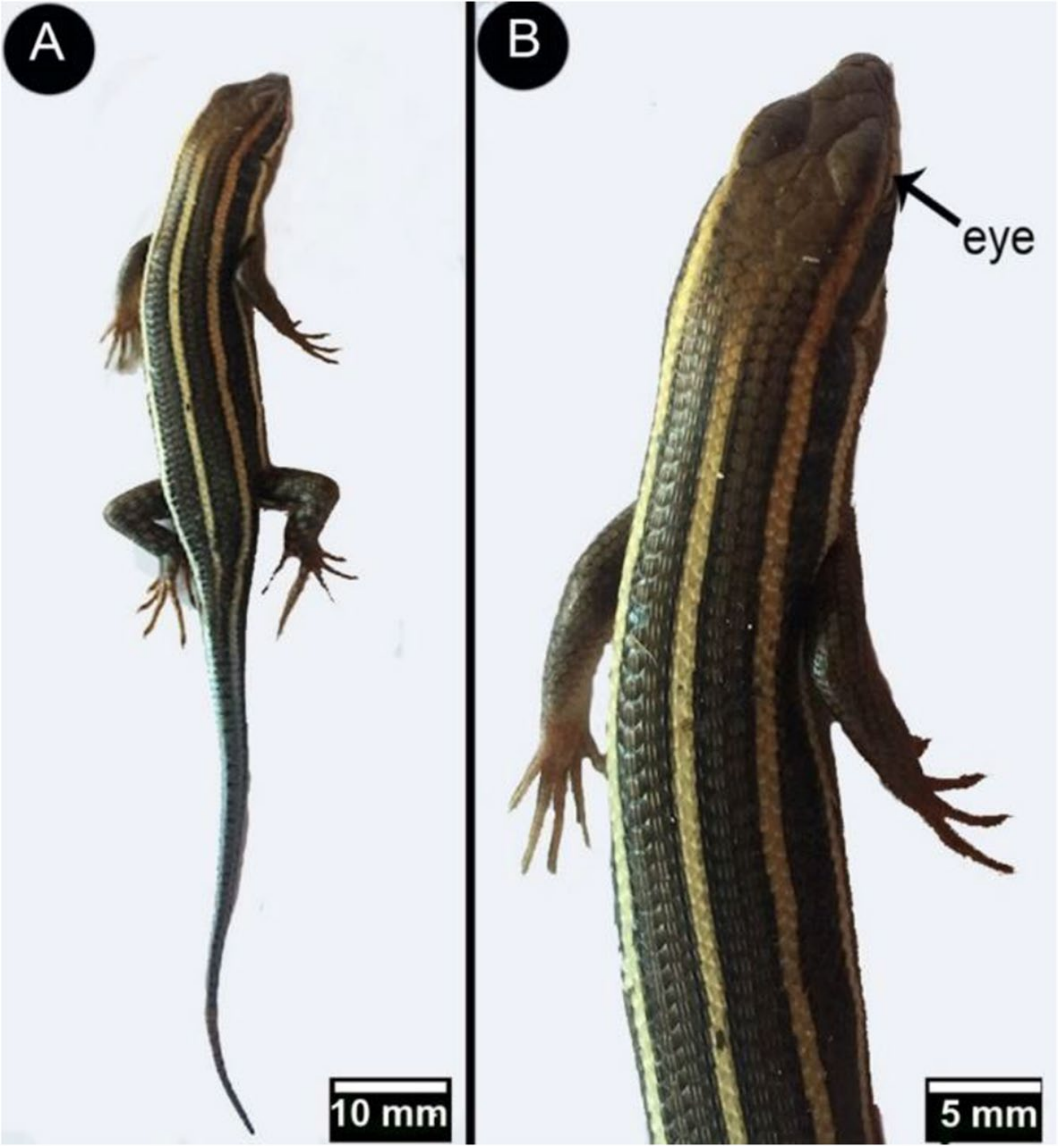
Overview images (View** A** by scale bar 10 mm) dorsal view and (View **B** by scale bar 5 mm) Enlarged part of the dorsal surface of the African five-lined skink

The African five-lined skink (*Trachylepis quinquetaeinata*) is a medium-sized lizard that measures 20 cm (7.9 inches). Its scales were prismatic and reflected metallic light. It was mostly olive brown or dark brown, with some shiny whitish spots and three olive-brown or dark brown stripes running from the head to the electric blue tail. The total axial length of the eye (from cranial to caudal part) was 3.972 ± 0.16 mm, and the transverse diameter was 4.85 ± 0.25 mm (Fig. 1A, B).

**Fig. 2:**
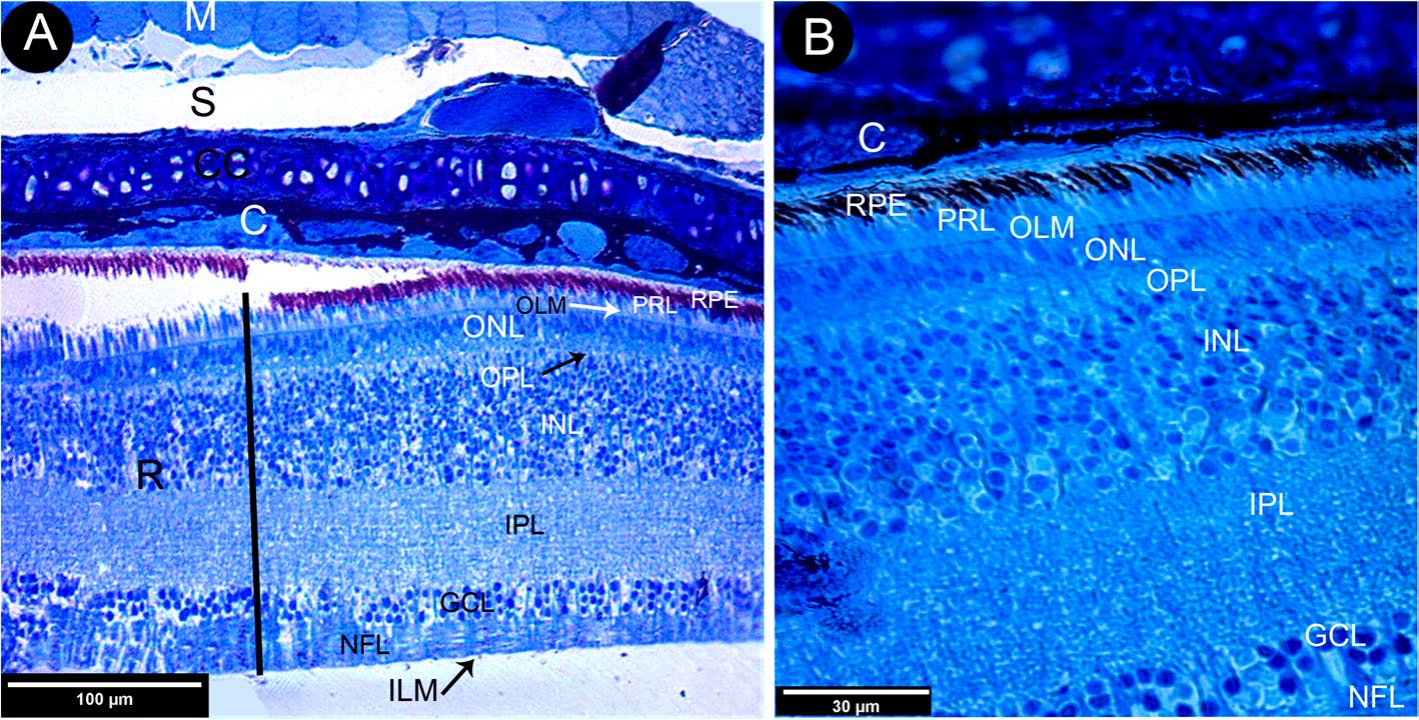
Photomicrographs of a transverse section of the eye of the African five-lined skink (View** A** toluidine blue × 100 by scale bar 100 µm) and enlarged transverse section of the retina (View **B** toluidine blue × 400 by scale bar 30 µm). They explained mainly the layers of retina, they showing the following structures, cartilaginous cup (CC) was located between choroid (C) and sclera (S) and retina(R) and the retinal layers were, retinal pigment epithelium (RPE), photoreceptor layer (PRL), outer limiting membrane (OLM), outer nuclear layer (ONL), outer plexiform layer (OPL), inner plexiform layer (INL), inner plexiform layer (IPL) ganglion cell layer (GCL), (NFL) nerve fiber layer and inner limiting membrane (ILM)

**Fig. 3:**
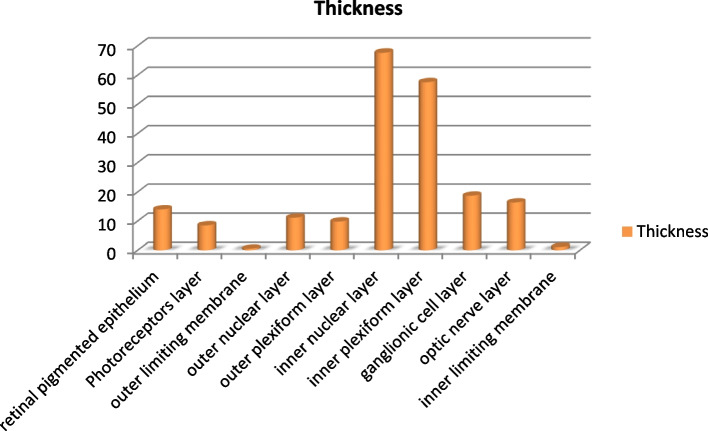
A chart explains the mean of the thickness of the layers of the retina of the African five-lined skink

The retina encased the choroid, which contained several wide venous sinuses that coalesced within melanin pigments. The retina's ten layers were identified (Fig. 2A, B). The thickness of the retinal layers was measured, where the inner nuclear layer was the thickest by an average of 67.66 ± 1.09 μm, followed by the inner plexiform layer by an average of 57.56 ± 0.94 μm, which maximizes the connectivity activity of the retinal neurons. The photoreceptor layer was 8.52 ± 0.50 μm considered the thinnest layer after the membranous layers of the retina, and the retinal pigmented epithelium was about 13.99 ± 0.44 μm (Table 1 and Fig. 3).

**Table 1:** Explains the mean of the thickness of the layers of the retina of the African five-lined skink

Layer of retina	Thickness (μm) Mean ± S.E
Retinal pigmented epithelium	13.99 ± 0.44
Photoreceptors layer	8.52 ± 0.50
Outer limiting membrane	0.54 ± 0.04
Outer nuclear layer	11.13 ± 0.4
Outer plexiform layer	9.82 ± 0.28
Inner nuclear layer	67.66 ± 1.09
Inner plexiform layer	57.56 ± 0.94
Ganglionic cell layer	18.69 ± 0.56
Optic nerve layer	16.37 ± 0.85
Inner limiting membrane	1.12 ± 0.11

**Fig. 4:**
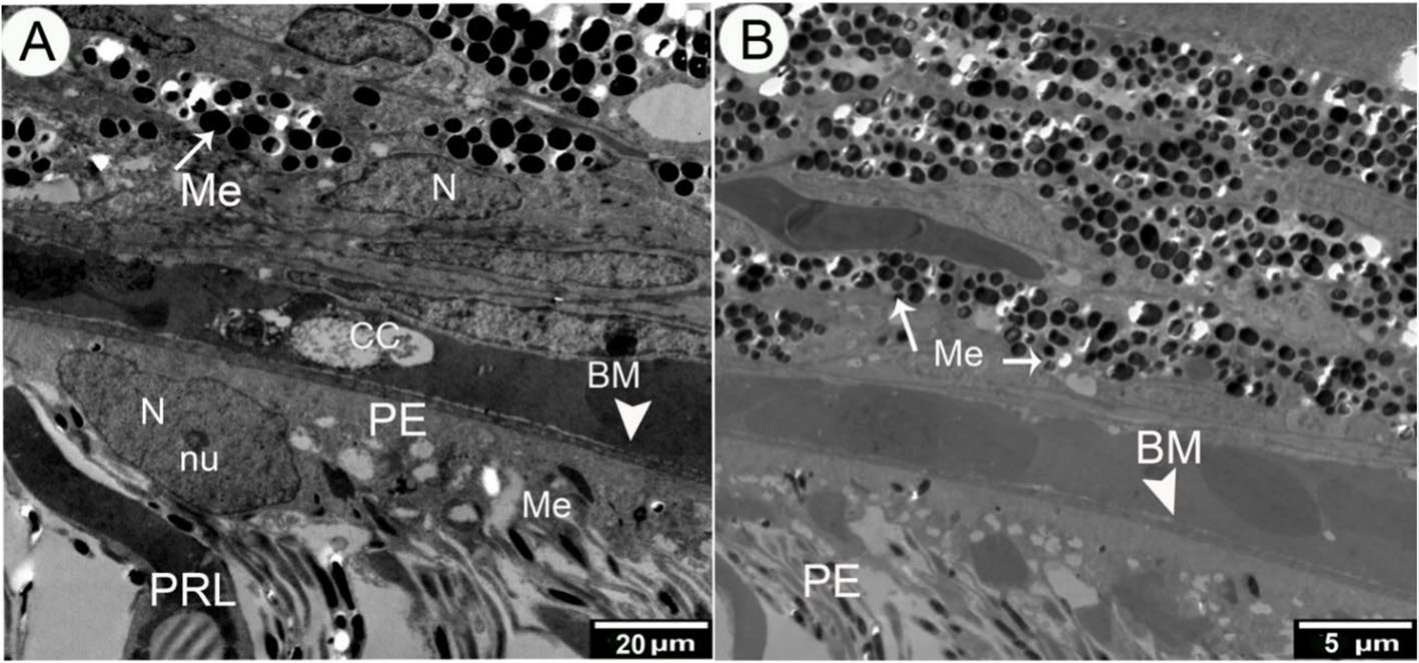
Transmission electron micrograph of the Retinal pigment layer of African five-lined skink. (View **A** Micro.Mag × 1200 by scale bar 20 µm & View **B** Micro.Mag × 2500 by scale bar 5 µm) explained the following structures: the choroid (CH) and pigment epithelial cell (PE) with their characteristic oval nucleus (N) and prominent nucleoli(nu). There are large number of variable shaped melanosomes (Me), choriocapillary layer (CC), Bruchʼ membrane (BM), and photoreceptor layer (PRL)

**Fig. 5:**
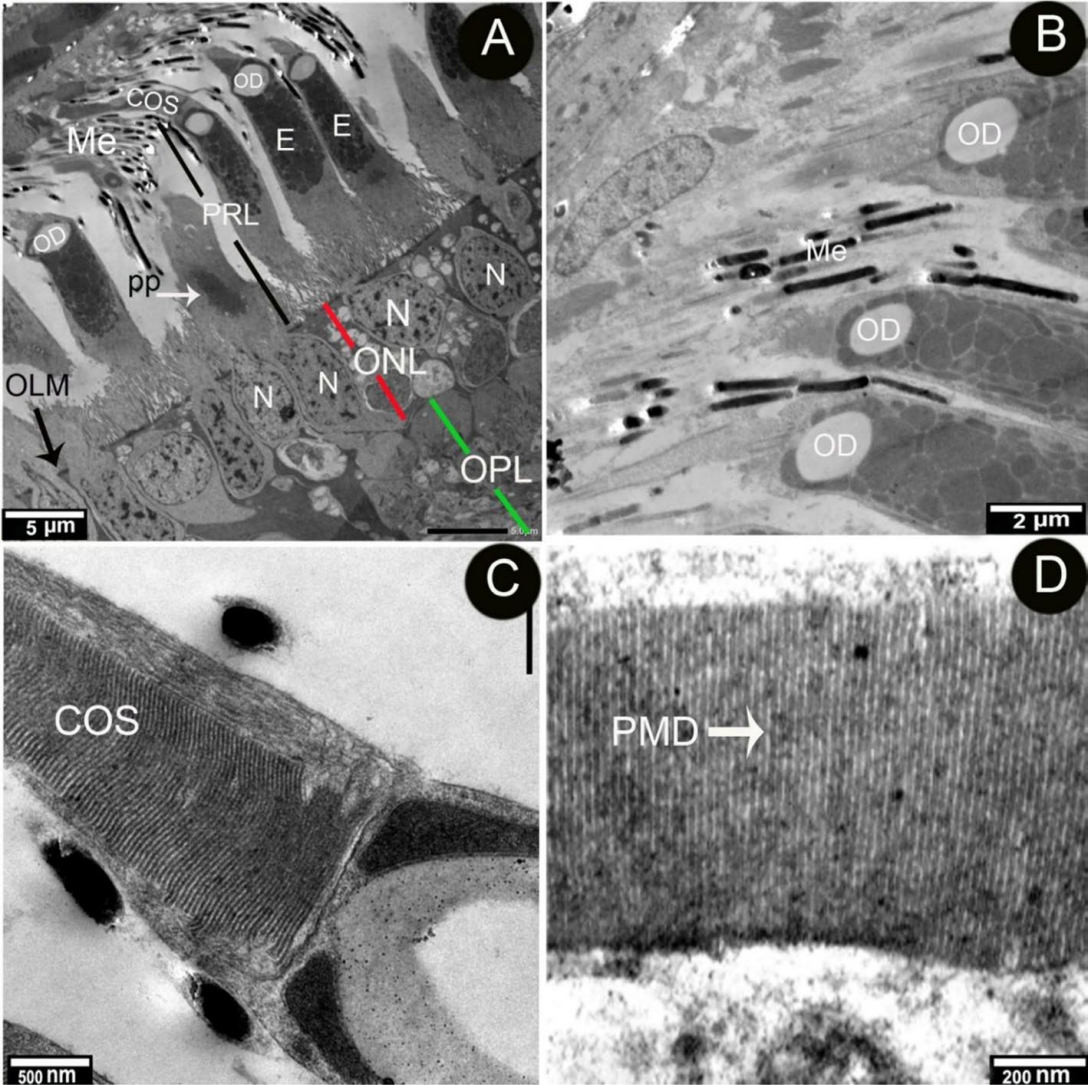
Transmission electron micrograph of the retinal pigment epithelium of the African five-lined skink extending into photoreceptor layer and containing large numbers of melanosomes. (View **A** Micro.Mag × 1000 by scale bar 5 µm & View **B** Micro.Mag × 3000 by scale bar 2 µm) and longitudinal section of the outer segment of photoreceptor with enlarged part. (View **C** Micro.Mag × 10,000 by scale bar 500 nm & View **D** Micro.Mag × 25,000 by scale bar 200 nm), They explained the following, photoreceptor layer (PRL), cone outer segment (COS), oil droplet (OD) ellipsoid (E), outer limiting membrane (OLM), Nucleus (N) outer nuclear layer, (ONL), outer plexiform layer (OPL), Melanosomes (Me), paraboloid, (PP), and parallel membranous disc (PMD)

The retinal pigment epithelium was composed of a single layer of cuboidal cells that lay in the Bruch's membrane, which separated it from the choroidal capillaries and choroidal melanosomes (which were mostly rounded in shape) (Figs. 2 and 4A,B). The microvillar processes of the retinal pigment epithelium interdigitated with the outer segment of the photoreceptor layer (Fig. 5A, B).

**Fig. 6:**
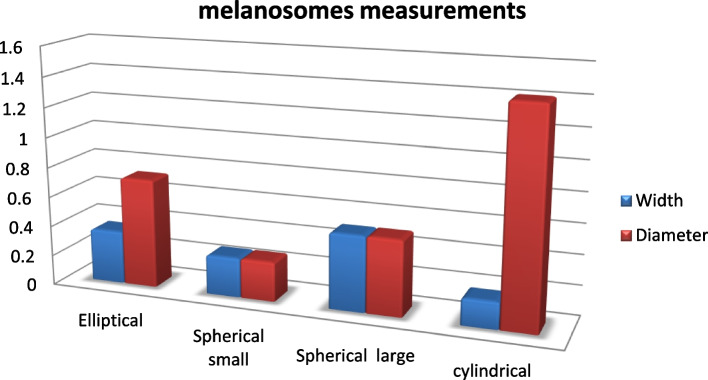
A chart explains the mean measurements of the melanosomes of the African five-lined skink

The RPE melanosomes were dark-shaped and had mainly elliptical and spheric melanosomes. The elliptical melanosomes measured 0.2–0.5 μm in width, with an average of 0.35 ± 0.07 μm, and0.4–0.9 μm in diameter, with an average of 0.07 ± 0.1 μm, and the spherical melanosomes measured 0.26 ± 0.02 μm in width, and 0.26 ± 0.01 μm in diameter, and the large was 0.50 ± 0.08 μm in width, and 0.50 ± 0.08 μm in diameter. The melanosomes in the apex of the cone were cylindrical 0.1–0.2 μm with an average of 0.18 ± 0.01 μm in width and 0.7–2.2 μm with an average of 1.4 ± 0.27 μm in diameter (Figs. 4A, B and 5A, B) (Table 2 and Fig. 6).

**Table 2:** Explains the shape and measurements of melanosomes of the thickness of the layers of the retina of the African five-lined skink

Melanosomes	Width (μm)		Diameter or length (μm)	
	**Range**	**Mean ± S.E**	**Range**	**Mean ± S.E**
**Elliptical**	0.2–0.5	0.35 ± 0.07	0.4–0.9	0.07 ± 0.1
**Small spherical**	0.2–0.3	0.26 ± 0.02	0.2–0.3	0.26 ± 0.01
**Large spherical**	0.4–0.6	0.50 ± 0.08	0.4–0.6	0.50 ± 0.08
**cylindrical**	0.1–0.2	0.18 ± 0.01	0.7–2.2	1.4 ± 0.27

The retina's photoreceptor layer was mostly made up of single cones with a few double cones. The double cone was made up of the principal and accessory cones. The cones had inner and outer segments separated by oil droplets. The inner segment was distinguished by the presence of an ellipsoid, a paraboloid, an outer fiber, a nucleus, and an inner fiber leading to a terminal synapse. The paraboloid was a somewhat spherical body that represented the area between the outer limiting membrane and the ellipsoid. The large paraboloid was only present in the accessory cone of the double cones. A paraboloid was present in all single cones (Fig. 5A).

The outer segments were made up of stacks of parallel membranous discs surrounded by a plasma membrane. All the outer segments were separated from one another by pigment epithelium processes containing elliptical melanosomes (Fig. 5B-D).

**Fig. 7:**
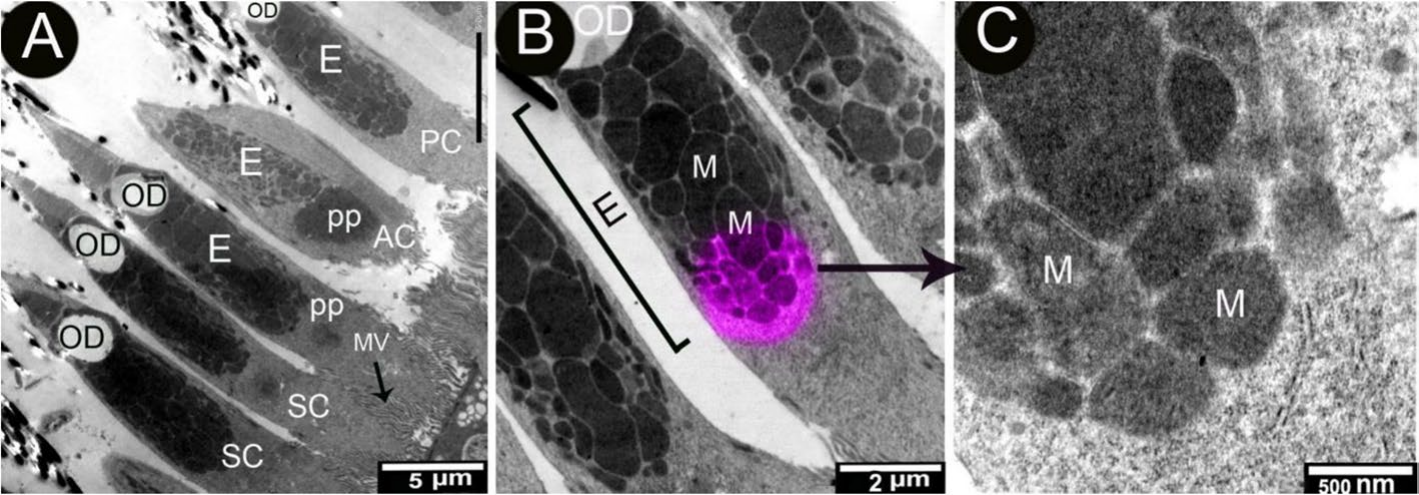
Transmission electron micrograph of longitudinal section through the double and single cones of the African five-lined skink (View **A** Micro.Mag × 1500 by scale bar 5 µm), Longitudinal section through ellipsoidal region of the photoreceptors (cone), (View **B** Micro.Mag × 4000 by scale bar 2 µm) and enlarged view of ellipsoid (View **C** Micro.Mag × 15,000 by scale bar 500 nm). They explained the major member of the double cone has oil droplet (OD), MV microvilli of Muller cell (MV), paraboloid (PP), ellipsoid(E), principal cone (pc), accessory cone (AC), single cone (SC), and mitochondria (M)

The oil droplets were found in the ellipsoid's proximal region. The oil droplets had plane boundaries and no discernible internal structure. They were partially bordered by the ellipsoid's mitochondrial outer membranes, particularly at the vitreal part of the ellipsoid. The mitochondria were larger in the center of the ellipsoid and smaller on the outside (Fig. 7). Oil droplets were observed in the single and double cones (Fig. 7A). Their lengths ranged from 1.33 to 2.3 μm, with a mean of 1.74 0.159 m, and their widths ranged from 0.96 to 1.9 um, with a mean of 1.308 ± 0.158 μm.

**Fig. 8:**
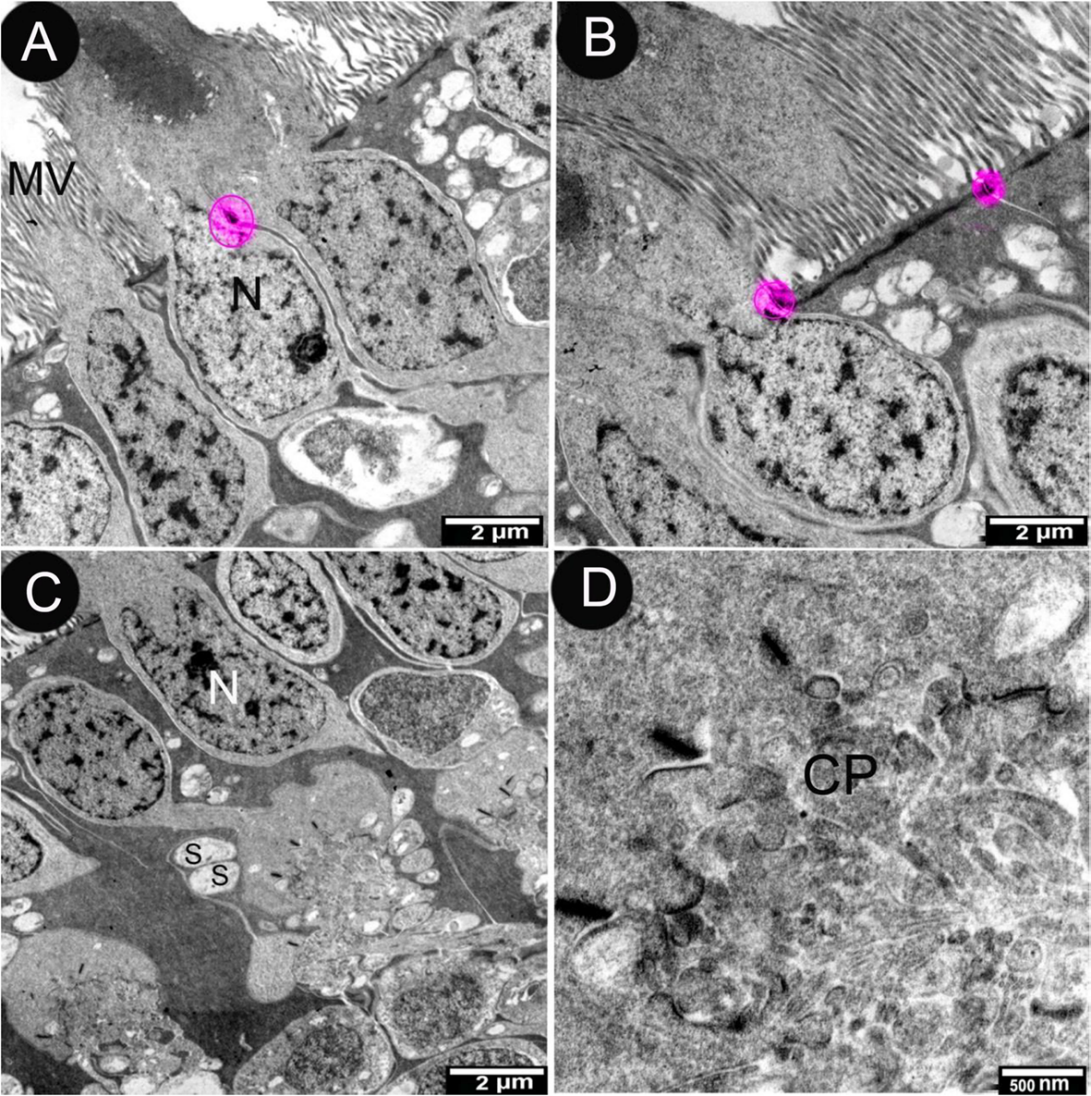
Transmission electron micrograph of the retina of the African five-lined skink showing the outer nuclear layer (View **A** Micro.Mag × 2000 by scale bar 2 µm & View **B** Micro.Mag × 4000 by scale bar 2 µm) and the outer nuclear layer and the outer plexiform layer (View **C** Micro.Mag × 2000 by scale bar 2 µm) and enlarged view of synaptic pedicle of the cone (View **D** Micro.Mag × 10,000 by scale bar 500 nm) they explained the nuclei (N) of the photoreceptor cells indicating that the cells of the retinal pigment epithelium adhere by tight junction (circle). microvilli (MV), synaptic terminal (S) of the cone, and one pedicle (CP)

Attachment areas between Müller's cells or between Müller's cells and photoreceptor cells made up the external limiting membrane. The dark and extended processes of Muller cell and zonula adherence were observed (Figs. 5A and 8A, B). The outer nuclear layer contained one row of visual cell nuclei. An external limiting membrane represented the nuclei, which were either circular or elongated (Fig. 8A-C).

The outer plexiform layers were thin and composed of horizontal and bipolar cell processes and cone pedicles (Fig. 8C). The pedicels were separated by Müller's cell extensions (Fig. 8C). The pedicels mostly contained round synaptic vesicles with a few synaptic ribbons (Fig. 8D). All synaptic terminals studied were lake mitochondria.

**Fig. 9:**
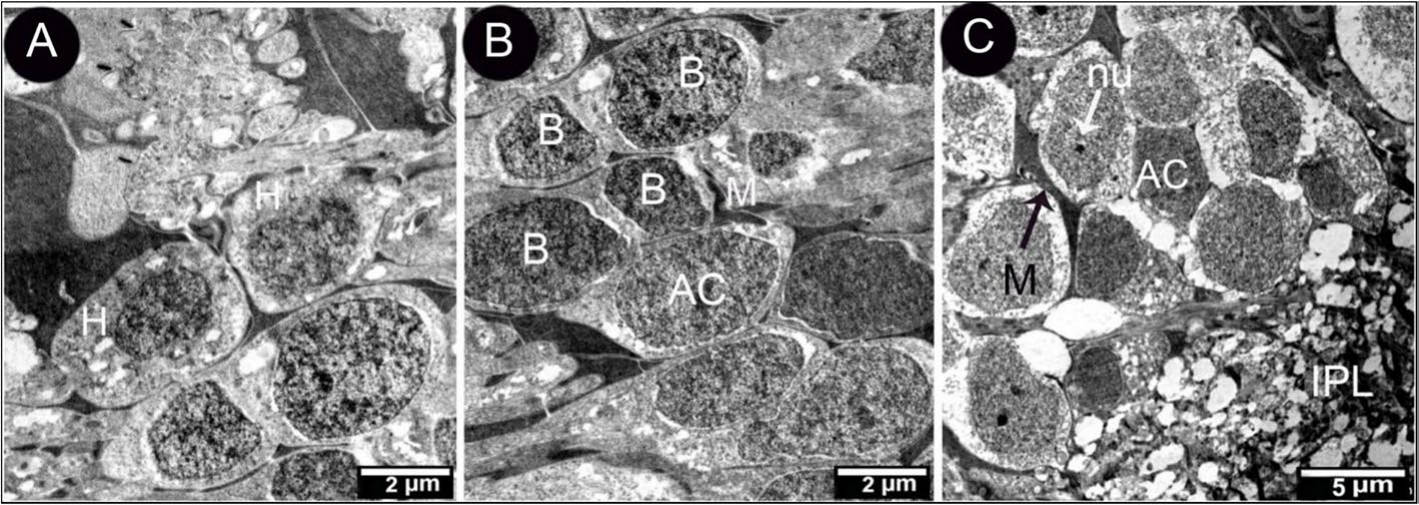
Transmission electron micrograph of the retina of the African five-lined skink at the outer plexiform and inner nuclear layer (View **A** Micro.Mag × 3000 by scale bar 2 µm), at the inner nuclear layer (View **B** Micro.Mag × 3000 by scale bar 2 µm), and at the inner nuclear layer and inner plexiform layer (View **C** Micro.Mag × 1500 by scale bar 5 µm), they showing the following plexiform and inner nuclear layer with horizontal cell (H). skink inner nuclear layer with bipolar (B), amacrine (AC) separated by processes of Muller cell whose nuclei (M) are present in the middle part of inner nuclear cell, the inner plexiform layer (IPL) with nuclei of amacrine (AC), muller cell nuclei (M), and another cell shown are bipolar cell nucleolus (nu)

Inner nuclear layers were more densely stained than the visual cell and the thickest layers. It had four to seven layers and four cell types: horizontal, bipolar, amacrine, and Muller cells (Fig. 9). The inner plexiform layer contained the horizontal cells with a large nucleus and clear cytoplasm, which sent dendrites to the OPL that they connected with photoreceptor terminals (Fig. 9A). Bipolar cells appeared between the horizontal and amacrine cells. Their cell bodies were either round or oval, with a round nucleus (Fig. 9B). Machine cells were distinguished from other cells by their location within the inner zone of INL (Fig. 9B). Müller cells constituted most glial cells in the retina. These cells extended radially and had a fusiform cell body with an oval nucleus located in the middle part of INL, their cytoplasm was very dark, and their processes penetrated in-between all the neurons (Fig. 9B-C).

**Fig. 10:**
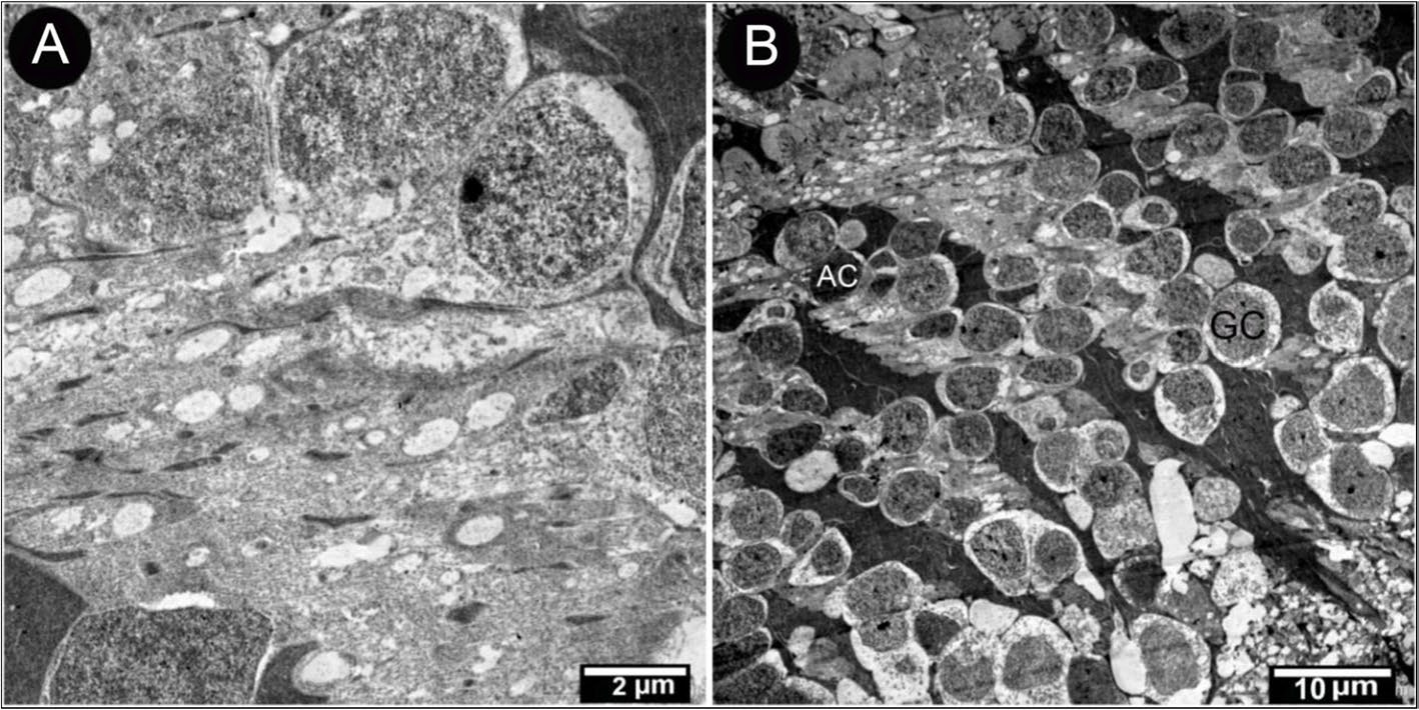
Transmission electron micrograph of the retina of the African five-lined skink inner plexiform layer. (View **A** Micro.Mag × 3000 by scale bar 2 µm) and the ganglion cell layer. (View **B** Micro.Mag × 600 by scale bar 10 µm) with ganglion (GC) and amacrine cells (AC)

The inner plexiform layer was composed of a complex meshwork of processes from neurons situated in the inner nuclear layer and the ganglion cell layer (Fig. 10A). The ganglion cell layer comprised of 2–3 rows of ganglion cells (Fig. 10B).

## Discussion

Diurnal animals are generally active in a photopic environment, whereas nocturnal animals are active in a scotopic climate. However, nighttime is not the only time when light is scarce. Many lizards are diurnal but use scotopic vision because they live in low-light environments such as burrows or under-leaf litter [22]. The current study discovered that the African five-lined skink (*Trachylepis quinquetaeniata*) has a specialized, diurnal-adapted retina. Our ultrastructure study revealed that the African five-lined skink had retinal adaptation to a day-light lifestyle. It had the potential for visual intensity, which could aid in catching mobile prey such as insects or butterflies.

The African five-lined skink's RPE contains numerous highly functional factories for lipid biosynthesis, which is required for vitamin A esterification and storage. The RPE includes many mitochondria and a smooth endoplasmic reticulum. Plentiful mitochondria indicate highly active RPE cells, implying a faster rate of transport and metabolism. Myeloid bodies are specialized endoplasmic reticulum components that appear to play a role in the metabolism of visual pigments. Histochemical detection of esterase activity confirmed them as lipid storage sites before esterification. These functional specializations emphasize the importance of RPE in metabolic insulation and how it assists the overlying neural retina in important functions such as vitamin A adjustment and storage, visual pigment renewal and synthesis, retinal adhesion maintenance, and photoreceptor waste phagocytosis. Furthermore, when exposed to visible or ultraviolet light, RPE melanosomes have been shown to catalyze free radical activity [6, 17, 30–33].

The presence of spherical and round melanosomes with smaller surface areas toward the direction of incoming light increased light access to the outer segments. In contrast to the presence of cylindrical melanosomes with larger surface areas on the cones' lateral surfaces, which increased light scattering and absorption [34].

We found only single and double cones in the retina of this African five-lined skink, which, like other diurnal lizards, contained only cones [7, 13, 35]. While the Australian species *Tiliqua rugosa* had a mixed retina with an 80:1 cone-to-rod ratio or a central retina ratio ranging from 20 to 40 cones for only one rod [5, 19].

According to the current ultrastructural findings, the cone's outer segment contained a strap of bimembranous discs where photopigments served as the light capture field for photoreceptors [6]. The inner segment of photoreceptor cells was known as the cell's synthetic center, where the material for new outer segment discs and other cellular functions was produced [36].

The inner segment of the photoreceptors of the African five-lined skink was distinguished by the presence of oil droplets, ellipsoids, and a paraboloid (a glycogen accumulation). Many lizard species' photoreceptors contained an inclusion known as an oil droplet, which was located either within or just distal to the ellipsoid. Oil droplets, as their name implies, are primarily composed of lipids and may also contain light-absorbing carotenoid pigments. Pigmented oil droplets can be found in the cones of birds, turtles, lizards, and lungfish, while colorless oil droplets can be found in some geckos, anuran amphibians, chondrostean fishes, marsupials, and monotremes. Oil droplets were found in single cones and the principal member of double cones in the African five-lined skink, as in other lizard species. Lampreys, teleosts, elasmobranchs, snakes, crocodilians, and placental mammals have no oil droplets [37]. Pigmented oil droplets act as filters, adjusting the photoreceptor's spectral sensitivity. Non-pigmented oil droplets, like liposomes, likely capture and focus light into the outer segment. Diurnal lizard photoreceptors contained oil droplets [38].

The ellipsoid mitochondria, numerous polytomies, RER, SER, Golgi zones, and autophagic vacuoles within the inner segment are signs of metabolic activity (Braekevelt, 1998). The high-density mitochondria created the large ellipsoid body seen at the apex of the photoreceptor inner segment, immediately vitreal to the oil droplet. Refractile organelles are found in many lizard species, and in addition to their function in cellular energetics, they can also collect light [7, 39]. The ellipsoid body of the African five-lined skink was arranged with large mitochondria in the center and smaller ones on the periphery. A scleral-vitreal-oriented gradient of mitochondrial size is found in diurnal geckos and other lizards but not in turtles or crocodilians [38]. Because of the ellipsoid body's proximity to the oil droplet and the interspersion of lipid microdroplets within snake ellipsoidal mitochondria [40], it has been proposed that the oil droplet is formed through transmutation [38] or secretion [41].

Pigmented oil droplets are represented in the cones of birds, turtles, lizards, and lungfish; colorless oil droplets occur in some geckos, anuran amphibians, and some monotremes [42, 43]. The existence of the oil droplets in the cones and the presence of spherical lipidic structures in the inner segments of cones gave them the important role of focusing light, much like spherical lenses [11]. The occurrence of the transparent oil droplets was a condition of ultraviolet senility, as had been explained in many species of birds [44]. The arrangement of the mitochondria on the ellipsoid part of each cone might play a role in concentrating light into the outer segment [45]. The ellipsoid mitochondria and paraboloid bodies, in addition to oil droplets, may acted as refractive and can accomplish a light-collecting role in addition to their function in cellular energetics [4, 38, 39].

A paraboloid body occurs in a subset of photoreceptor cells in all reptiles except snakes [38]. The organelle consists of granules of glycogen and a tubular membrane continuous with the rough endoplasmic reticulum, and between species varies to extremes of membranous or granular [41]. The paraboloid bodies observed in *Ctenophorus ornatus* are of the granular type. As in *Ctenophorus ornatus*, reptiles have a paraboloid in all single cones and one member of the double cones. As for the ellipsoid body, the paraboloid is involved with cellular metabolism and the transport of macromolecules through the synthesis and storage of glycogen [38]. The organelle may also be involved with light collection; it is absent only from the principal member of the double cones, in which the paraboloid of the accessory member is contained within the path of incident light [38].

The OLM in African five-lined skink, like in other vertebrate species, was formed by a series of zonulae adherents between photoreceptors and Muller cells. Landolt's club has been described in several species as a ciliated dendrite of a bipolar cell that projects through the ELM [15]. These clubs have no known function and were found in a different elasmobranch, the southern fiddler ray (*Trygonorhina fasciata*) [15], but not in the short-tailed stingray. Muller cells projected numerous short fingerlike processes through the OLM of many species, including the short-tailed stingray. These surrounded the photoreceptor inner segments at their base, while their function is unknown, they are thought to be important in exchange functions because they are more numerous in avascular retinas [5].

In the present work, the inner nuclear layer was the thickest and ranged from 6–8 layers a somewhat the thick inner nuclear layer had eight to nine cell layers characteristic of the retinae of strictly diurnal vertebrates. Nocturnal animals tend to have a considerably thinner inner nuclear layer. Remarkably, there was a conspicuous difference in the thickness of the inner nuclear layer of the retina [4].

## Conclusions

The retina of the African five-lined skink exhibited ultrastructural characteristics related to its diurnal lifestyle in its environment. Long cylindrical melanosomes with a larger surface area on the lateral surfaces of the cones were demonstrated. The cones contained elliptical to ovoid oil droplets with variable diameters, which reduced chromatic aberration and improved color while potentially focusing light onto the cone segments. The presence of double cones increased the available light absorption area. The retina contained glycogen deposits (paraboloids), which acted as both an adaptation to visual acuity and a source of energy for the visual cells.

## Data Availability

The datasets used and/or analyzed during the current study are available from the corresponding author on reasonable request.

## Abbreviations

TEM: Transmission Electron Microscopy
RPE: Retinal Pigment Epithelium
OPL: Outer Plexiform Layer
INL: Inner Nuclear Layer
RER: Rough Endoplasmic Reticulum
SER: Smooth Endoplasmic Reticulum
OLM: Outer Limiting Membrane
